# Pyrochlore-Supergroup Minerals Nomenclature: An Update

**DOI:** 10.3389/fchem.2021.713368

**Published:** 2021-09-06

**Authors:** Daniel Atencio

**Affiliations:** Instituto de Geociências – Universidade de São Paulo, São Paulo, Brazil

**Keywords:** pyrochlore supergroup, nomenclature, pyrochlore group, microlite group, elsmoreite group, roméite group

## Abstract

The general formula of the pyrochlore-supergroup minerals is *A*_2_ *B*_2_ *X*_6_ *Y*. The mineral names are composed of two prefixes and one root name (identical to the name of the group). The first prefix refers to the dominant anion (or cation or H_2_O or vacancy) of the dominant valence at the *Y*-site. The second prefix refers to the dominant cation of the dominant valence [or H_2_O or vacancy] at the *A*-site. Thirty-one pyrochlore-supergroup mineral species are currently distributed into four groups [pyrochlore (*B* = Nb, *X* = O), microlite (*B* = Ta, *X* = O), roméite (*B* = Sb^5+^, *X* = O), and elsmoreite (*B* = W, *X* = O)] and two unassigned members [hydrokenoralstonite (*B* = Al, *X* = F) and fluornatrocoulsellite (*B* = Mg, *X* = F)]. However, when the new nomenclature system of this supergroup was introduced (2010) only seven mineral species, namely, oxycalciopyrochlore, hydropyrochlore, hydroxykenomicrolite, oxystannomicrolite, oxystibiomicrolite, hydroxycalcioroméite, and hydrokenoelsmoreite, were valid. The seven species belong to the cubic crystal system and space group *Fd* $\bar{3}$ *m* and O is predominant in the *X* structural site. The 24 new mineral species described between 2010 and 2021 are cesiokenopyrochlore, fluorcalciopyrochlore, fluornatropyrochlore, hydrokenopyrochlore, hydroxycalciopyrochlore, hydroxynatropyrochlore, hydroxykenopyrochlore, hydroxymanganopyrochlore, hydroxyplumbopyrochlore, fluorcalciomicrolite, fluornatromicrolite, hydrokenomicrolite, hydroxycalciomicrolite, kenoplumbomicrolite, oxynatromicrolite, oxycalciomicrolite, oxybismutomicrolite, fluorcalcioroméite, hydroxyferroroméite, oxycalcioroméite, oxyplumboroméite, fluornatrocoulsellite, hydrokenoralstonite, and hydroxykenoelsmoreite. Among the new species, hydroxycalciomicrolite belongs to a different space group of the cubic system, i.e., *P*4_2_32. There are also some mineral species that crystallize in the trigonal system. Hydrokenoelsmoreite occurs as 3*C* (*Fd* $\bar{3}$ *m*) and 6*R* (*R* $\bar{3}$) polytypes. Hydrokenomicrolite occurs as 3*C* (*Fd* $\bar{3}$ *m*) and 3*R* (*R* $\bar{3}$ *m*) polytypes, of which the latter corresponds to the discredited “parabariomicrolite.” Fluornatrocoulsellite crystallizes as 3*R* (*R* $\bar{3}$ *m*) polytype. Surely there are several new pyrochlore-supergroup minerals to be described.

## Introduction

The nomenclature system currently valid for the pyrochlore supergroup was introduced by Atencio et al. (2010) to replace the one authored by Hogarth (1977). Subsequently, clarifications (Christy and Atencio, 2013), remarks (Hogarth, 2013), a response to the remarks (Atencio, 2013), and a paper on the incorporation of two minerals already known to the supergroup (Atencio et al., 2017) were published. When the new nomenclature system of this supergroup was introduced (Atencio et al., 2010), only seven mineral species, namely, oxycalciopyrochlore, hydropyrochlore, hydroxykenomicrolite, oxystannomicrolite, oxystibiomicrolite, hydroxycalcioroméite, and hydrokenoelsmoreite, were valid. Between 2010 and 2021, 24 new mineral species were described. The following text describes the nomenclature of pyrochlore-supergroup minerals. The nomenclature system has been updated to include the pyrochlore-supergroup minerals discovered in recent years, whose available information might be difficult for an interested reader to find. The representative minerals from each group are discussed in detail. All known minerals of the supergroup are listed in one place, so this text can be regarded as a kind of a digest of all natural species. There are two aims in compiling this data. The first is to enable a reader to identify both the chemical composition and source of a given mineral and the second is to enable the reader to identify the primary data associated with the mineral.

## Crystallography, Chemistry, and the Nomenclature Scheme

The general formula of the pyrochlore-supergroup minerals is *A*_2_ *B*_2_ *X*_6_ *Y*. In this formula, *A* typically is a large [8]-coordinated cation with a radius of ∼1.0 Å or a vacancy (□) but can also be H_2_O. For structural reasons, *A* can be subdivided into constituents without lone-pair electrons (*e.g.*, Na, Ca), which occupy 16 d in *Fd* $\bar{3}$ *m*, and stereoactive cations (e.g., Sb^3+^), which occupy less symmetrical positions displaced slightly from 16d, e.g., 96 g. For the purpose of this nomenclature, no subdivision is made. The *A*-site therefore may host Na, Ca, Ag, Mn, Sr, Ba, Fe^2+^, Pb^2+^, Sn^2+^, Sb^3+^, Bi^3+^, Y, Ce (and other *REE*), Sc, U, Th, □, or H_2_O. *B* is a [6]-coordinated cation (site 16c), typically of high field-strength. This site thus may contain Ta, Nb, Ti^4+^, Sb^5+^, W, but also V^5+^, Sn^4+^, Zr, Hf, Fe^3+^, Mg, Al, and Si. *X* typically is O but can include subordinate OH and F (site 48f). *Y* typically is an anion but can also be a vacancy, H_2_O, or a very large (>>1.0 Å) monovalent cation (site 8b). Examples are OH^−^, F, O, □, H_2_O, K, Cs, and Rb. Displacements to 96g, 32e, and 192i positions were also located. See the basis for formula calculation in Atencio et al. (2010). Synthetic pyrochlores have a much more variable chemical composition than natural examples (Subramanian et al., 1983).

The mineral names are composed of two prefixes and one root name (identical to the name of the group). The first prefix refers to the dominant anion (or cation or H_2_O or □) of the dominant valence at the *Y*-site. The second prefix refers to the dominant cation of the dominant valence [or H_2_O or □] at the *A*-site. Where the first and second prefixes are equal, then only one prefix is applied (“hydropyrochlore,” not “hydrohydropyrochlore”). The mineral groups are given in Table 1.

**TABLE 1:** Mineral groups of the pyrochlore supergroup.

Group	*B*	*X*
Elsmoreite	W^6+^	O^2-^
Pyrochlore	Nb^5+^	O^2-^
Microlite	Ta^5+^	O^2-^
Roméite	Sb^5+^	O^2-^
Betafite	Ti^4+^	O^2-^
Ralstonite	Al^3+^	F^1-^
Coulsellite	Mg^2+^	F^1-^

*B*: the dominant cation of the dominant valence at the *B*-site.

*X*: the dominant anion of the dominant valence at the *X*-site.

As a mineral group consists of two or more minerals (Mills et al., 2009), ralstonite and coulsellite cannot really be considered, for now, as mineral groups. Hydrokenoralstonite and fluornatrocoulsellite should be designated as unassigned members of the pyrochlore supergroup, because there is no other member to allow a group to be established.

Currently, there is no valid betafite-group mineral.

**FIGURE 1:**
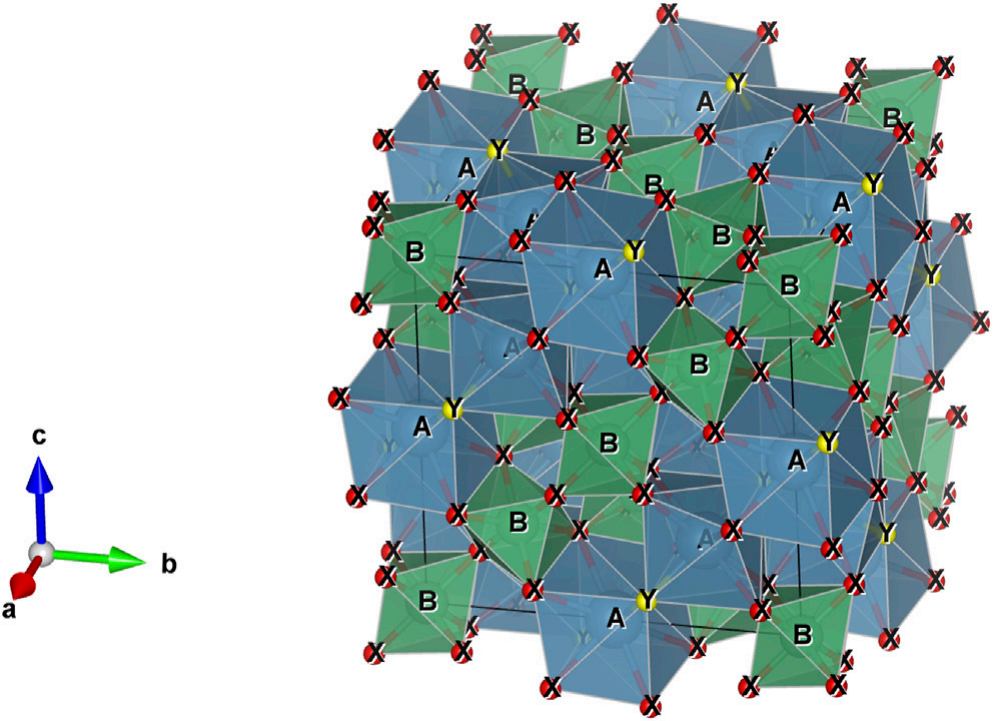
Pyrochlore crystal structure drawn using VESTA 3 (Momma and Izumi, 2011).

The seven species valid in 2010 belong to the cubic crystal system and space group *Fd* $\bar{3}$ *m.* Among the new species, hydroxycalciomicrolite belongs to a different space group of the cubic system, i.e., *P*4_2_32. There are also some mineral species that crystallize in the trigonal system. Hydrokenoelsmoreite occurs as 3*C* (*Fd* $\bar{3}$ *m*) and 6*R* (*R* $\bar{3}$) polytypes. Hydrokenomicrolite occurs as 3*C* (*Fd* $\bar{3}$ *m*) and 3*R* (*R* $\bar{3}$ *m*) polytypes, of which the latter corresponds to the discredited “parabariomicrolite.” Fluornatrocoulsellite crystallizes as 3*R* (*R* $\bar{3}$ *m*) polytype. The symmetry is lowered due to ordering on either A sites or B sites (Ercit et al., 1986; Rouse et al., 1998; Atencio, 2016; Mills et al., 2016; Andrade et al., 2017; Mills et al., 2017). The pyrochlore structure (Figure 1) is an essential building block for other minerals and mineral groups, such as alunite (Goreaud and Raveau, 1980) or pittongite (Grey et al., 2006).

## The Groups and Species

Table 2 shows the species of the pyrochlore supergroup, except hydrokenoralstonite and fluornatrocoulsellite.

**TABLE 2:** Mineral species of the pyrochlore (P), microlite (M), roméite (R), elsmoreite (E), and betafite (B) groups. Blue: already valid in 2010; red: expected in 2010 and described later; brown: expected in 2010 but not yet described; green: not foreseen in 2010 but described later.

*A*↓ *Y*→	O	OH	F	□	H_2_O	Cs
□		P M E	P		P M E	P
H_2_O					P M	
Na	M P	P	P M R			
Ca	P M R B	P M R	P M R			
Sr			P			
Fe^2+^		R				
Mn^2+^		P				
Sn^2+^	M					
Pb	P R	P		P M		
Sb^3+^	M					
Bi	M					
Y	P					
U	B					

Following that, simplified formulae are given for the pyrochlore species. Note that subordinate components at the *A*, *B*, *X*, or *Y* sites have no nomenclatural significance and any of these could be replaced by “#”, indicating an unspecified heterovalent species required for charge balance.

## Pyrochlore Group

**Oxycalciopyrochlore** (Atencio et al., 2010), Ca_2_Nb_2_O_6_O, *Fd* $\bar{3}$ *m, a* 10.356(2) Å, *V* 1110.6 (7) Å^3^, first described by Černý et al. (1979) as “stibiobetafite.” The IMA number was not quoted in the original description of “stibiobetafite.” Type locality: Věžná I pegmatite, Věžná, Žďár nad Sázavou District, Vysočina Region, Czech Republic.

**Hydropyrochlore** (Atencio et al., 2010), (H_2_O, #)_2_Nb_2_O_6_(H_2_O), *Fd* $\bar{3}$ *m, a* 10.580 Å, *V* 1184.29 Å^3^, first described by van Wambeke (1978) as “kalipyrochlore.” The IMA number was not quoted in the original description of “kalipyrochlore.” Type locality: Lueshe Mine, Bwito, Rutshuru Territory, North Kivu, DR Congo.

**Cesiokenopyrochlore** (Agakhanov et al., 2021), (□, #)_2_Nb_2_O_6_Cs, *Fd* $\bar{3}$ *m, a* 10.444(1) Å, *V* 1139.5(2) Å^3^. IMA number: 2016-104. Type locality: Tetezantsio pegmatites, Tetezantsio-Andoabatokely Pegmatite Field, Andrembesoa, Betafo, Vakinankaratra, Madagascar.

**Fluorcalciopyrochlore** (Li et al., 2016), (Ca, #)_2_Nb_2_O_6_F, *Fd* $\bar{3}$ *m, a* 10.4164(9) Å, *V* 1130.19 Å^3^. IMA number: 2013-055. Type locality: Bayan Obo deposit (Bayun-Obo deposit; Baiyunebo deposit), Bayan Obo, Bayan Obo mining district, Baotou City (Baotou Prefecture), Inner Mongolia, China.

**Fluornatropyrochlore** (Jingwu et al., 2015), (Na, #)_2_Nb_2_O_6_F, *Fd* $\bar{3}$ *m, a* 10. 5,053(10) Å, *V* 1159.4 Å^3^. IMA number: 2013-056. Type locality: Boziguoer intrusion, Baicheng Co. (Bay Co.), Akesu Prefecture (Aksu Prefecture; Aqsu Prefecture), Xinjiang, China.

**Hydrokenopyrochlore** (Biagioni et al., 2018), (□, #)_2_Nb_2_O_6_(H_2_O), *Fd* $\bar{3}$ *m, a* 10.4887(8) Å, *V* 1153.9 Å^3^. IMA number: 2017-005. Type locality: Antandrokomby pegmatite, Manandona Valley, Sahatsiho Ambohimanjaka, Ambositra, Amoron’i Mania, Madagascar.

**Hydroxycalciopyrochlore** (Yang et al., 2014), (Ca, #)_2_Nb_2_O_6_(OH), *Fd* $\bar{3}$ *m, a* 10.381(4) Å, *V* 1118.7 Å^3^. IMA number: 2011-026. Type locality: Maoniuping Mine, Mianning County, Liangshan Yi, Sichuan, China.

**Hydroxynatropyrochlore** (Ivanyuk et al., 2019), (Na, #)_2_Nb_2_O_6_(OH), *Fd* $\bar{3}$ *m, a* 10.3276(5) Å, *V* 1101.5 Å^3^. IMA number: 2017-074. Type locality: Phoscorite-carbonatite pipe, Kovdor Massif, Murmansk Oblast, Russia.

**Hydroxykenopyrochlore** (Miyawaki et al., 2017, pending publication), (□, #)_2_Nb_2_O_6_ (OH), *Fd* $\bar{3}$ *m, a* 10.590(5) Å, *V* 1187.65 Å^3^. IMA number: 2017-030a. Type locality: Araxá mine, Araxá complex, Barreiro, Araxá, Minas Gerais, Brazil.

**Hydroxymanganopyrochlore** (Chukanov et al., 2013), (Mn^2+^, #)_2_Nb_2_O_6_ (OH), *Fd* $\bar{3}$ *m, a* 10.2523(2) Å, *V* 1077.62 Å^3^. IMA number: 2012-005. Type locality: In den Dellen quarries, Mendig, Mendig, Mayen-Koblenz District, Rhineland-Palatinate, Germany.

**Hydroxyplumbopyrochlore** (Li et al., 2020), (Pb, #)_2_Nb_2_O_6_ (OH), *Fd* $\bar{3}$ *m, a* 10.558 (2) Å, *V* 1176.91 Å^3^. IMA number: 2018-145. Type locality: Jabal Sayid mine (Jabal Sayid Cu-Zn deposit), Medina Region, Saudi Arabia.

**“Fluorstrontiopyrochlore”** (Atencio et al., 2010), (Sr, #)_2_Nb_2_O_6_F, a possible new species, analysis published (Franchini et al., 2005). Occurrence: Jasimampa prospect, Ojo de Agua Department, Santiago del Estero Province, Argentina.

**“Fluorkenopyrochlore”** (Atencio et al., 2010), (□, #)_2_Nb_2_O_6_F, a possible new species, analyses published (Kartashov et al., 1998; Schmitt et al., 2002). Occurrences: Khaldzan Buragtag massif, Myangad District, Khovd Province, Mongolia; Amis Complex, Brandberg Complex, Brandberg Area, Dâures Constituency, Erongo Region, Greenland.

**“Oxynatropyrochlore”** (Atencio et al., 2010), (Na, #)_2_Nb_2_O_6_O, a possible new species, analyses published (Hogarth and Horne 1989; Knudsen 1989; Chukanov et al., 1999). Occurrences: Locality 2, Ndale Area, Fort Portal, Kabarole, Western Region, Uganda; Qaqqaarsuk, Maniitsoq, Maniitsoq Island, Qeqqata, Greenland; Mika pegmatite, Rangkul' Highlands, Gorno-Badakhshan, Tajikistan.

**“Oxyplumbopyrochlore”** (Atencio et al., 2010), Pb_2_Nb_2_O_6_O, a possible new species, analysis published (Voloshin and Pakhomovskiy, 1986). Occurrence: Kola Peninsula, Murmansk Oblast, Russia.

**“Oxyyttropyrochlore-(Y)”** (Atencio et al., 2010), (Y, #)_2_Nb_2_O_6_O, a possible new species, analysis published (Tindle and Breaks, 1998). Occurrence: Separation Rapids Lithium Project (Separation Lake area), Kenora District, Ontario, Canada.

**“Kenoplumbopyrochlore”** (Atencio et al., 2010), (Pb, #)_2_ Nb_2_O_6_□, a possible new species, analysis published (Voloshin and Pakhomovskiy, 1986). Occurrence: Ploskaya Mt, Western Keivy Massif, Keivy Mountains, Lovozersky District, Murmansk Oblast, Russia.

## Microlite Group

**Hydroxykenomicrolite** (Atencio et al., 2010), (□, #)_2_Ta_2_O_6_ (OH), *Fd* $\bar{3}$ *m, a* 10.526 (5) Å, *V* 1166.244 Å^3^, first described by Voloshin et al. (1981) as “cesstibtantite.” The IMA number was not quoted in the original description of “cesstibtantite.” Type locality: Vasin-Myl’k Mt, Voron’i Tundry, Murmansk Oblast, Russia.

**Oxystannomicrolite** (Atencio et al., 2010), Sn_2_Ta_2_O_6_O, *Fd* $\bar{3}$ *m, a* 10.470 Å, *V* 1147.73 Å^3^, first described by Vorma and Siivola (1967) as “sukulaite.” The IMA number was not quoted in the original description of “sukulaite.” Type locality: Sukula Pegmatites, Tammela, Tavastia Proper, Finland.

**Oxystibiomicrolite** (Atencio et al., 2010), (Sb^3+^, #)_2_Ta_2_O_6_O, *Fd* $\bar{3}$ *m, a* 10.455(2) Å, *V* 1142.80 Å^3^, first described by Groat et al. (1987) as “stibiomicrolite.” The IMA number was not quoted in the original description of “stibiomicrolite.” Type locality: Varuträsk, Skellefteå, Västerbotten County, Sweden.

**Fluorcalciomicrolite** (Andrade et al., 2013a), (Ca^2+^, #)_2_ Ta_2_O_6_F, *Fd* $\bar{3}$ *m, a* 10.4191 (6) Å, *V* 1131.07 Å^3^. IMA number: 2012-036. Type locality: Volta Grande mine (Mibra mine), Nazareno, Minas Gerais, Brazil.

**Fluornatromicrolite** (Witzke et al., 2011), (Na, #)_2_ Ta_2_O_6_F, *Fd* $\bar{3}$ *m, a* 10.4451 (2) Å, *V* 1139.56 Å^3^. IMA number: 1998-018. Type locality: Quixaba pegmatite, Quixaba, Frei Martinho, Paraíba, Brazil.

**Hydrokenomicrolite** (Andrade et al., 2013b; Atencio, 2016), (□, #)_2_Ta_2_O_6_ (H_2_O). Hydrokenomicrolite-3*C* polytype: Cubic, *Fd* $\bar{3}$ *m, a* 10.454(1) Å. *V* 1142.5 (2) Å^3^. Hydrokenomicrolite-3*R* polytype: Trigonal, *R* $\bar{3}$ *m, a* 7.4290(6), *c* 18.505 (2) Å, *V* 884.5 (1) Å^3^. IMA Numbers: hydrokenomicrolite (hydrokenomicrolite-3*C*) 2011-103; “parabariomicrolite” (hydrokenomicrolite-3*R*): 84-3. Type localities: Hydrokenomicrolite-3*C* (described as hydrokenomicrolite by Andrade et al., 2013b), Volta Grande pegmatite, Nazareno, Minas Gerais, Brazil. Hydrokenomicrolite-3*R* (formerly described as “parabariomicrolite” by Ercit et al., 1986), Alto do Giz pegmatite, Equador Co., Rio Grande do Norte, Brazil.

**Hydroxycalciomicrolite** (Andrade et al., 2017), (Ca^2+^, #)_2_ Ta_2_O_6_(OH), *P*4_2_32, *a* 10.4205(8) Å *V* 1131.53 Å^3^. The first pyrochlore-supergroup mineral with long range ordering of Ca and □ on the *A* sites, that invokes reduction of symmetry. IMA number: 2013-073. Type locality: Volta Grande mine (Mibra mine), Nazareno, Minas Gerais, Brazil.

**Kenoplumbomicrolite** (Atencio et al., 2018)**,** (Pb, #)_2_ Ta_2_O_6_□, *P*4_2_32, *a* 10.575 (5) Å *V* 1182.6 Å^3^. IMA number: 2015-007-a. Type locality: Ploskaya Mt, Western Keivy Massif, Keivy Mountains, Lovozersky District, Murmansk Oblast, Russia.

**Oxynatromicrolite** (Fan et al., 2016), (Na, #)_2_ Ta_2_O_6_O, *Fd* $\bar{3}$ *m, a* 10.420(6) Å, *V* 1131.4 Å^3^. IMA number: 2013-063. Type locality: Pegmatite vein no. 309, Guanpo pegmatite field, Guanpo, Lushi County, Sanmenxia, Henan, China.

**Oxycalciomicrolite** (Menezes da Silva et al., 2020), Ca_2_Ta_2_O_6_O, *Fd* $\bar{3}$ *m, a* 10.4325 (4) Å, *V* 1135.46(14) Å^3^. IMA number: 2019-110. Type locality: Fumal pegmatite, Nazareno, Minas Gerais, Brazil.

**Oxybismutomicrolite** (Kasatkin et al., 2020), (Bi, #)_2_ Ta_2_O_6_O, *Fd* $\bar{3}$ *m, a* 10.475 (1) Å, *V* 1149.38 Å^3^. IMA number: 2019-047. Type locality: Solnechnaya pegmatite, Malkhan pegmatite field (Malchan; “Malechansk”), Krasnyi Chikoy, Zabaykalsky Krai, Russia.

**“Hydromicrolite”** (Atencio et al., 2010), (H_2_O, #)_2_ Nb_2_O_6_(H_2_O), a possible new species, analysis published (Andrade, 2007). Occurrence: Volta Grande mine (Mibra mine), Nazareno, Minas Gerais, Brazil.

## Roméite Group

**Hydroxycalcioroméite** (Atencio et al., 2010), (Ca, #)_2_Sb^5+^_2_O_6_(OH), *Fd* $\bar{3}$ *m, a* 10.264 Å, *V* 1081.31 Å^3^, first described by Hussak and Prior (1895) as “lewisite.” IMA number: a pre-IMA mineral. Type locality: Tripuí (Tripuhy), Ouro Preto, Minas Gerais, Brazil.

**Fluorcalcioroméite** (Atencio et al., 2013), (Ca, #)_2_ Sb^5+^_2_O_6_F, *Fd* $\bar{3}$ *m, a* 10.2987 (8) Å, *V* 1092.31 Å^3^. IMA number: 2012-093. Type locality: Starlera Mine, Starlera Valley, Ferrera, Viamala Region, Grisons, Switzerland.

**Hydroxyferroroméite** (Mills et al., 2017a), (Fe^2+^, #)_2_Sb^5+^_2_O_6_(OH), *Fd* $\bar{3}$ *m, a* 10.25 (3) Å, *V* 1077 Å^3^. IMA number: 2016-006. Type locality: Correc d’en Llinassos (Ravin d’en Llinassous), Oms, Céret, Pyrénées-Orientales, Occitanie, France.

**Oxycalcioroméite** (Biagioni et al., 2013), Ca_2_Sb^5+^_2_O_6_O, *Fd* $\bar{3}$ *m, a* 10.3042 (7) Å, *V* 1094.06 Å^3^. IMA number: 2012-022. Type locality: Buca della Vena Mine, Ponte Stazzemese, Stazzema, Lucca Province, Tuscany, Italy.

**Oxyplumboroméite** (Hålenius and Bosi, 2013), Pb_2_Sb^5+^_2_O_6_O, *Fd* $\bar{3}$ *m, a* 10.3783 (6) Å, *V* 1117.84 Å^3^. IMA number: 2013-042. Type locality: Harstigen Mine, Pajsberg, Persberg ore district, Filipstad, Värmland County, Sweden.

**“Fluornatroroméite”** (Atencio et al., 2010), (Na, #)_2_ Sb^5+^_2_O_6_F, a possible new species, crystal structure determined (Matsubara et al., 1996). Occurrence: Gozaisho mine, Iwaki, Japan.

## Elsmoreite Group

**Hydrokenoelsmoreite** (Atencio et al., 2010), □_2_W_2_O_6_ (H_2_O), first described by Williams et al. (2005) as “elsmoreite”. Hydrokenoelsmoreite-3*C* polytype: Cubic, *Fd* $\bar{3}$ *m, a* 10.3065(3) Å. *V* 1094.80 Å^3^. Hydrokenoelsmoreite-6*R* polytype: Trigonal, *R* $\bar{3}$ *, a* 7.2882(2), *c* 35.7056(14) Å, *V* 1642.51 Å^3^. IMA Numbers: “elsmoreite” (hydrokenoelsmoreite-3*C*) 2003-059. Type localities: Hydrokenoelsmoreite-3*C* (described as elsmoreite by Williams et al., 2005): Elsmore Tin Mine (Elsmore Tin lodes), Elsmore, Gough Co., New South Wales, Australia; hydrokenoelsmoreite-3*C* and hydrokenoelsmoreite-6*R* (formerly “ferritungstite”) from Hemerdon mine (now Drakelands mine) in Devon, United Kingdom (Mills et al., 2016).

**Hydroxykenoelsmoreite** (Mills et al., 2017b), (□, #)_2_ W_2_O_6_(OH), Trigonal, *R* $\bar{3}$ *, a* 7.313(2), *c* 17.863(7) Å, *V* 827(1) Å^3^. IMA number: 2016-056. Type locality: Masaka gold mine, Muyinga Province, Burundi.

## Unassigned Members

**Fluornatrocoulsellite** (Atencio et al., 2017), (Na, #)_2_ Mg_2_F_6_F, *R* $\bar{3}$ *m, a* 7.1620(1), *c* 17.5972(3) Å, *V* 781.7049 Å^3^, first described by Birch et al. (2009) as “cousellite.” IMA number: 2009-046 (coulsellite). Type locality: Mt Cleveland Mine, Luina, Heazlewood district, Waratah-Wynyard municipality, Tasmania, Australia.

**Hydrokenoralstonite** (Atencio et al., 2017), □_2_Al_2_F_6_(H_2_O), *Fd* $\bar{3}$ *m, a* 9.91(4) Å. *V* 973.24 Å^3^, first described by Brush (1871) as “ralstonite.” IMA number: pre-IMA mineral. Type locality: Ivigtut Mine, Arsuk Fjord, Sermersooq, Greenland.

## Betafite Group

**“Oxycalciobetafite”** (Atencio et al., 2010), (Ca,#)_2_Ti_2_O_6_O, a possible new species, analysis published (Meyer and Yang, 1988). Occurrence: Fra Mauro Base (Apollo 14 landing site), Fra Mauro Highlands, The Moon.

**“Oxyuranobetafite”** (Atencio et al., 2010), (U, #)_2_ Ti_2_O_6_O, a possible new species, analysis published (Mokhov et al., 2008). Occurrence: Luna 24 landing site, Mare Crisium, The Moon.

## Data Availability

The original contributions presented in the study are included in the article/supplementary material; further inquiries can be directed to the corresponding author.
